# Borrelidins C–E: New Antibacterial Macrolides from a Saltern-Derived Halophilic *Nocardiopsis* sp.

**DOI:** 10.3390/md15060166

**Published:** 2017-06-06

**Authors:** Jungwoo Kim, Daniel Shin, Seong-Hwan Kim, Wanki Park, Yoonho Shin, Won Kyung Kim, Sang Kook Lee, Ki-Bong Oh, Jongheon Shin, Dong-Chan Oh

**Affiliations:** 1 Natural Products Research Institute, College of Pharmacy, Seoul National University, Seoul 08826, Korea; xyrho25@gmail.com (J.K.); pharmcraft87@snu.ac.kr (D.S.); yanberk@snu.ac.kr (S.-H.K.); dicafree@korea.com (Y.S.); pooh6764@snu.ac.kr (W.K.K.); sklee61@snu.ac.kr (S.K.L.); shinj@snu.ac.kr (J.S.); 2 Department of Agricultural Biotechnology, College of Agriculture and Life Sciences, Seoul National University, Seoul 08826, Korea; adamas2001@hanmail.net (W.P.); ohkibong@snu.ac.kr (K.-B.O.)

**Keywords:** antibacterial, saltern, halophilic actinomycetes, borrelidin, *Salmonella enterica*

## Abstract

Chemical investigation of a halophilic actinomycete strain belonging to the genus *Nocardiopsis* inhabiting a hypersaline saltern led to the discovery of new 18-membered macrolides with nitrile functionality, borrelidins C–E (**1**–**3**), along with a previously reported borrelidin (**4**). The planar structures of borrelidins C–E, which are new members of the rare borrelidin class of antibiotics, were elucidated by NMR, mass, IR, and UV spectroscopic analyses. The configurations of borrelidines C–E were determined by the interpretation of ROESY NMR spectra, *J*-based configuration analysis, a modified Mosher’s method, and CD spectroscopic analysis. Borrelidins C and D displayed inhibitory activity, particularly against the Gram-negative pathogen *Salmonella enterica*, and moderate cytotoxicity against the SNU638 and K562 carcinoma cell lines.

## 1. Introduction

Chemical studies of marine-derived actinomycetes in the past 20 years have resulted in the discovery of structurally and biologically diverse secondary metabolites [1,2]. Salinity is obviously a distinct factor that continuously promotes the metabolic adaptation of marine-derived actinomycetes to marine environments [3]. Salterns are the most extreme habitats with respect to salinity and can harbor truly halophilic actinomycetes [4]. However, the chemistry of actinobacteria in these hypersaline environments had been mostly neglected until our report of the salternamides as the first secondary metabolites from a saltern-derived actinomycete (*Streptomyces* sp.) in 2015 [5,6]. A subsequent biological study of salternamide A revealed that this chlorinated metabolite suppresses the hypoxia-induced accumulation of HIF-1*α* and induces apoptosis in human cancer cells [7]. Our continuing study of saltern-derived actinomycetes led to the discovery of indolosesquiterpenoids, called xiamycins C–E, from a halophilic *Streptomyces* sp. Xiamycin D displayed potent antiviral activity against porcine epidemic diarrhea virus (PEDV) [8]. These promising results indicated that saltern-derived actinobacteria can be utilized as prolific sources of bioactive secondary metabolites with pharmaceutical potential, leading us to expand our research program from the common genus *Streptomyces* to diverse phylogenetic groups in the phylum Actinobacteria that inhabit salterns.

**Figure 1:**
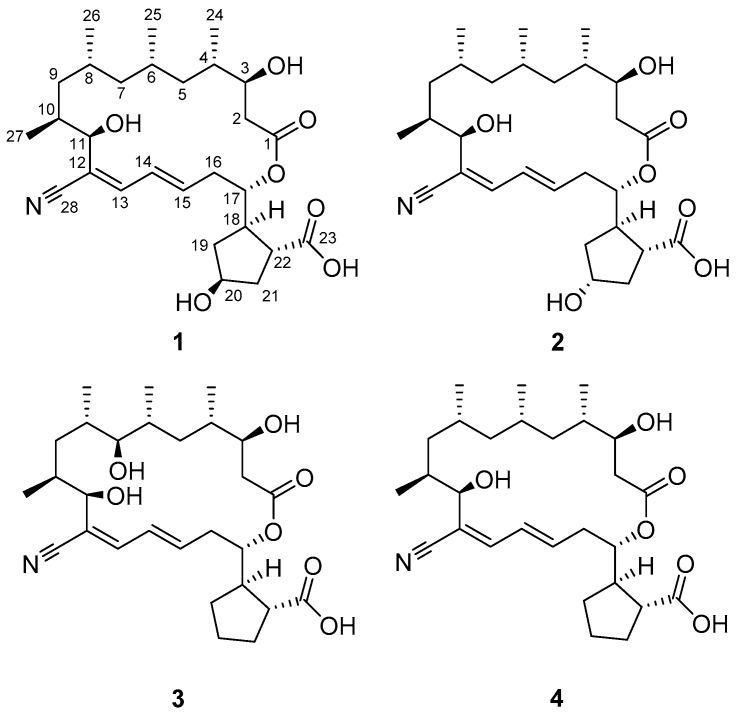
The structures of borrelidins C–E (**1**–**3**) and borrelidin (**4**).

Halophilic actinomycete strains were selectively isolated from topsoil samples collected from a saltern located in Jeungdo, Jeollanam-do, Republic of Korea. From among the isolated actinomycete strains, strain HYJ128, belonging to the genus *Nocardiopsis*, was cultivated in liquid medium. Chemical profiling based on an LC/MS analysis indicated that this strain produces a series of secondary metabolites that commonly display a UV spectrum with an absorption maximum at *λ*_max_ 257 nm, are expected to incorporate a conjugation of at least two double bonds and have an [M − H]^−^ molecular ion at *m*/*z* 504. This initial evaluation by UV spectroscopy and MS prompted the production of large cultures and chromatographic purification to obtain three new polyketide-derived macrolides, borrelidins C–E (**1**–**3**), which are new members of the borrelidin class of antibiotics, originally isolated from *Streptomyces* spp. [9] (Figure 1). Herein, we report the structures of borrelidins C–E from the rare halophilic actinomycete *Nocardiopsis* sp. HYJ128, along with the antibacterial and cytotoxic biological activities of these compounds. 

## 2. Results

### 2.1. Structural Elucidation

Borrelidin C (**1**) was obtained as a white powder that was determined to have the molecular formula C_28_H_43_NO_7_ with an unsaturation number of eight based on the analysis of negative ion mode high-resolution fast atom bombardment (HR-FAB) mass spectrometry data (obsd. [M − H]^−^ at *m*/*z* 504.2959, calcd. [M − H]^−^ 504.2961). The ^1^H NMR spectrum of **1** in pyridine-*d*_5_ displayed 3 double-bond protons (*δ*_H_ 6.83, 6.66, and 6.33), 4 oxymethine protons (*δ*_H_ 5.65, 4.79, 4.57, and 4.37), 20 aliphatic methine or methylene protons (*δ*_H_ 3.44~0.95), and 12 protons in four methyl groups (*δ*_H_ 1.28, 0.97, 0.91, and 0.85; Table 1). In the ^13^C NMR spectrum, 2 carbonyl carbons (*δ*_C_ 179.4 and 172.4), 5 resonances in the double bond region (*δ*_C_ 143.7, 139.1, 127.7, 120.1, and 118.3), 4 oxygen-bearing carbons (*δ*_C_ 76.7, 72.4, 72.2, and 70.8), and 17 aliphatic carbons (*δ*_C_ 48.3~15.4) were identified. The interpretation of the HSQC spectrum assigned all the ^13^C-^1^H one-bond correlations. Because the number of carbons in the double bond region was five and the molecular weight was an odd number, one of the five carbons between 118.3 and 143.7 should be associated with a nitrogen atom as an imine or nitrile functional group. An absorption peak at 2218 cm^−1^ in the IR spectrum and a ^13^C chemical shift of 118.3 ppm were observed, characteristically supporting the existence of nitrile functionality [10]. This nitrile functional group explains two of the eight double bond equivalents inherent in the molecular formula. In addition, the two carbonyl groups and two double bonds accounted for the unsaturation number of 4, thus revealing that borrelidin C (**1**) is a bicyclic compound. 

**Figure 2:**
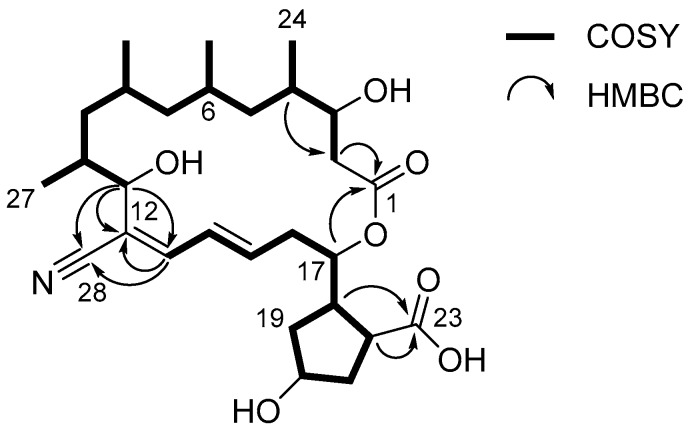
The key COSY and HMBC correlations of borrelidin C (**1**).

Examination of the ^1^H-^1^H COSY NMR spectrum of borrelidin C (**1**) identified two spin systems (Figure 2). H_2_-2 methylene began the first spin system by connecting C-2 and C-3 with a H_2_-2/H-3 COSY correlation. This chain was sequentially extended to C-11 by consecutive ^1^H-^1^H couplings from H-3 to H-11. Four branched methyl groups—C-24, C-25, C-26, and C-27—were respectively assigned at C-4, C-6, C-8, and C-10 by their COSY correlations with the corresponding methine protons. Second, H-13 (*δ*_H_ 6.83) displayed a correlation with H-14 at 6.66 ppm. H-14 extended the spin system to H-15 (*δ*_H_ 6.33) by a three-bond ^1^H-^1^H coupling between H-14 and H-15, thus constructing a diene moiety. The H-15/H_2_-16 (*δ*_H_ 2.60 and 2.57) COSY correlation extended the chain structure from the diene moiety to the aliphatic methylene C-16 (*δ*_C_ 36.2). A homonuclear correlation between H_2_-16 and H-17 (*δ*_H_ 5.65) connected the oxymethine C-17 (*δ*_C_ 76.7) to C-16. Additionally, an array of COSY correlations was observed from H-17 to H-22 through consecutive methylenes H_2_-19, H_2_-20, and H_2_-21, identifying a cyclopentane and extending the carbon framework from C-17 to C-22 (*δ*_C_ 48.3).

Despite the comprehensive analysis of the COSY NMR spectrum of **1**, two carbonyl carbons (*δ*_C_ 179.4 and 172.4), one olefinic carbon (*δ*_C_ 120.1), and a nitrile carbon (*δ*_C_ 118.3) were not assigned. These unassigned moieties were connected to the first and/or second spin systems by HMBC correlations. The C-1 carbonyl carbon (*δ*_C_ 172.4) was assigned next to C-2 (*δ*_C_ 39.3) by an HMBC correlation from H_2_-2 to C-1. The other carbonyl carbon (C-23) correlated with H-18 (*δ*_H_ 3.22) and H-22 (*δ*_H_ 3.44) in the HMBC NMR spectrum, confirming connectivity of this carbon to C-22 (*δ*_C_ 48.3). The olefinic quaternary carbon C-12 at 120.1 ppm was located between C-11 (*δ*_C_ 72.2) and C-13 (*δ*_C_ 143.7), thus linking the two spin systems based on H-11/C-12 and H-13/C-12 HMBC correlations. This connectivity was further secured by an HMBC correlation from H-11 (*δ*_H_ 4.57) to C-13. The nitrile functional group was assigned to C-12 based on three-bond heteronuclear couplings from H-11 and H-13 to the nitrile carbon C-28 (*δ*_C_ 118.3). Finally, a macrocyclic lactone ring was constructed based on a key HMBC correlation from H-17 (*δ*_H_ 5.65) to C-1, assigning C-1 as an ester carbon and subsequently proposing the C-23 carboxylic acid functional group. Therefore, the remaining two double bond equivalents of borrelidin C (**1**) were explained by the cyclopentane and the macrocyclic ring, and the planar structure of borrelidin C (**1**) was elucidated as a new member of the borrelidin class. Comparison of the structure of **1** with that of borrelidin (**4**) revealed that borrelidin C (**1**) has an additional hydroxyl group at C-20 in the cyclopentane ring.

Borrelidin D (**2**) was purified as a white powder with the molecular formula C_28_H_43_NO_7_ based on the analysis of HR-FAB-MS (obsd. [M − H]^−^ at *m*/*z* 504.2958, calcd. [M − H]^−^ 504.2961). The IR, UV, and mass spectra of this compound were analogous to those of borrelidin C (**1**). Because the ^1^H and ^13^C NMR and COSY, HSQC, HMBC, and ROESY NMR spectra were also very similar to those of **1**, the planar structure of borrelidin D (**2**) could be readily elucidated as a structure identical to that of borrelidin C (**1**) based on these NMR spectra, as shown in Figure 1. A careful analysis of the NMR spectra of **2** indicated that the difference between **1** and **2** could originate from the stereochemistry of the hydroxyl group at C-20 (*δ*_C_ 73.3) because the ^1^H and ^13^C chemical shifts of the cyclopentane exhibited noticeable differences.

Borrelidin E (**3**) was isolated as a white powder. The molecular formula was deduced as C_28_H_43_NO_7_ based on the HR-FAB-MS analysis (obsd. [M − H]^−^ at *m*/*z* 504.2966, calcd. [M − H]^−^ 504.2961). Because the spectroscopic data—including the 1D and 2D NMR, mass, UV, and IR spectra—displayed analogous features to those of borrelidins C and D (**1**–**2**), the planar structure of borrelidin E (**3**) could be determined by comparing the spectra with those of **1** and **2**. In the ^13^C NMR spectrum of **3**, a relatively deshielded oxygen-bearing carbon was detected at 81.5 ppm, which was not observed in borrelidins C and D. A comprehensive analysis of the 1D and 2D NMR spectra revealed that this oxygenated carbon was at C-7 (*δ*_C_ 81.5), not C-20 (*δ*_C_ 26.2), which is different from borrelidins C and D. Therefore, the structure of borrelidin E was established as a new congener of borrelidin with a hydroxyl group at C-7. 

**Figure 3:**
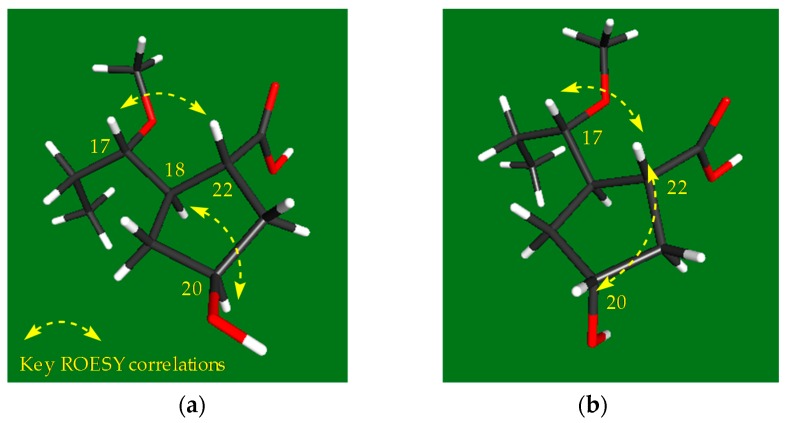
Key ROESY correlations around the cyclopentane of borrelidins C-D (**1**–**2**).

The configurations of the stereogenic centers of borrelidins C–E (**1**–**3**) were deduced to be identical to those of borrelidin (**4**) based on the high degree of similarity of the NMR and CD spectroscopic data (Figure S28) and the common biosynthetic origin of **1**–**4**. However, the configurations of the additional hydroxyl groups at C-20 in **1**–**2** and at C-7 in **3** required further analysis. In the ROESY NMR spectrum of borrelidin C (**1**), the H-17/H-22 correlation indicated that these protons are on the same face. More importantly, the H-18/H-20 correlation showed a *syn*-configuration between H-18 and H-20 on the face opposite from H-17 and H-22, thus establishing the relative configuration of the cyclopentane (Figure 3a). In contrast, in the ROESY NMR data of borrelidin D (**2**), H-20 correlated with H-22, which in turn correlated with H-17, demonstrating that these three protons are facing the same direction (Figure 3b). Therefore, the relative configuration of C-20 in borrelidin D was opposite to that in borrelidin C as expected vide supra.

**Figure 4:**
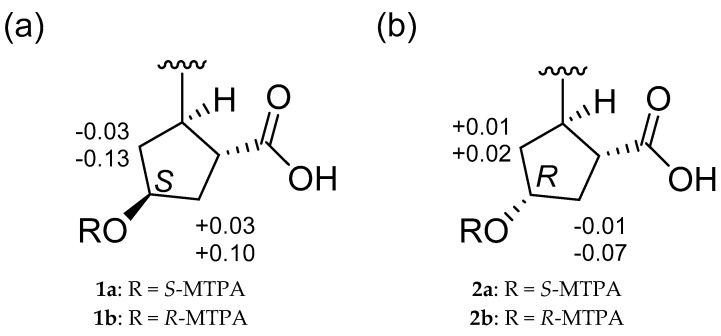
*Δδ_S-R_* values in ppm of (**a**) **1a** and **2b** and (**b**) **2a** and **2b** in pyridine-*d*_5_.

The absolute configuration of C-20 in borrelidin C (**1**) was determined by a modified Mosher’s method [11]. Bis-*S*- and *R*-MTPA esters (**1a** and **1b**) were obtained by derivatizing borrelidin C with *R*- and *S*-*α*-methoxy-*α*-(trifluoromethyl)phenylacetyl chloride (MTPA-Cl), respectively. Fortunately, the hydroxyl group at C-20 in **1** was successfully esterified with MTPA, thus enabling an analysis of the ^1^H chemical shifts. The ^1^H chemical shifts of **1a** and **1b** were assigned by analyzing their COSY NMR spectra. The *Δδ_S-R_* values of the ^1^H chemical shifts around the stereogenic center (C-20) were calculated (Figure 4a). The consistent distribution of the *Δδ_S-R_* value signs finally determined the 20*S* configuration. The absolute configuration of C-20 in borrelidin D (**2**) was also established by the modified Mosher’s method. As expected based on the relative configuration, the absolute configuration of C-20 in **2** was determined as *R*, which is opposite to that of C-20 in **1** (Figure 4b).

**Figure 5:**
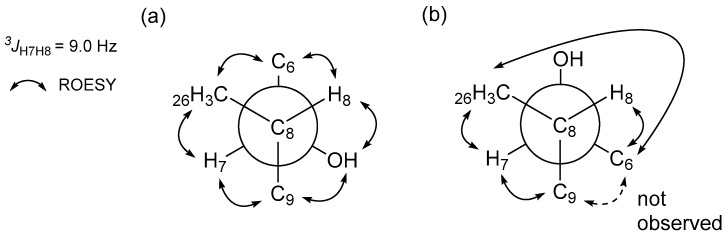
*J*-based configuration analysis for the configuration of borrelidin E (**3**) at C-7 and C-8. Two rotamers were selected with ^3^*J*_H7H8_ (9.0 Hz). (**a**) The rotamer satisfies the observed ROESY correlations; (**b**) The rotamer cannot satisfy the observed H-6/H_3_-26 ROESY correlation.

To investigate the configuration of C-7 in borrelidin E (**3**), a homo-decoupling ^1^H experiment was performed to obtain the ^1^H-^1^H coupling constants of H-7 (*δ*_H_ 3.26) and H-8 (*δ*_H_ 2.01), which were not clearly measurable in the ordinary ^1^H NMR spectrum because H-7 (broad doublet) coupled with H-6 as well. When H-6 at 2.38 ppm was irradiated, the coupling constant between H-7 and H-8 was successfully measured as 9.0 Hz (Figure S27). The *anti*-relationship between H-7 and H-8 was assigned based on this large ^3^*J*_H7H8_ value in the *J*-based configuration analysis (Figure 5) [12]. Further analysis of ROESY correlations with this H-7/H-8 *anti*-relationship enabled us to select of the correct rotamer and assign its 7*S* configuration based on the previously-established 8*S* configuration in borrelidin (**4**) [9] (Figure 5a). 

### 2.2. Bioactivities of the Borrelidins

Because borrelidin was initially reported as an antibacterial compound, the bioactivities of the new borrelidin family members (**1**–**3**) and borrelidin (**4**) were first evaluated in antimicrobial assays against phylogenetically diverse pathogenic bacterial strains, including *Staphylococcus aureus* ATCC 25923, *Enterococcus faecalis* ATCC 19433, *Enterococcus faecium* ATCC 19434, *Proteus hauseri* NRBC 3851, *Klebsiella pneumoniae* ATCC 10031, *Salmonella enterica* ATCC 14028, and *Escherichia coli* ATCC 25922, using ampicillin as a positive control compound (Table 2). Borrelidins C and D (**1**–**2**) displayed moderate antibacterial activity specifically against *S. enterica* ATCC 14028 with minimum inhibitory concentration (MIC) values of 16 µM and 63 µM, respectively, while borrelidin (**4**) broadly inhibited most of the tested pathogens except *S. aureus* and *E. coli*. Borrelidin (**4**) was also most active against *S. enterica*, with an MIC value of 0.51 µM, which is approximately three times more potent than the positive control ampicillin (MIC = 1.4 µM). However, borrelidin E (**3**) did not exhibit significant inhibitory activity against the tested bacteria. These results revealed that hydroxylation at C-20 significantly reduces antibacterial activity, and hydroxylation at C-7 can even abolish the activity. The specific inhibitory activities of **1**–**4** against *S. enterica* indicated that the borrelidin class might be a particularly useful scaffold for antibiotics against *S. enterica*, which causes salmonellosis mainly via food contamination [13]. 

Additionally, the cytotoxicities of borrelidins C–E (**1**–**3**) and borrelidin (**4**) against various human carcinoma cell lines—such as A549 (lung cancer), HCT116 (colon cancer), SNU638 (stomach cancer), SK-HEP1 (liver cancer), MDA-MB231 (breast cancer), and K562 (leukemia)—were measured (Table 3). Borrelidins C and D (**1**–**2**) showed significant cytotoxicity against the tested cancer cell lines. In particular, borrelidin C (**1**) exhibited considerable cytotoxic activity against SNU638 and K562 with IC_50_ values of 5.5 μM and 5.7 μM, respectively. Borrelidin D (**2**) displayed comparable cytotoxicity against these cancer cell lines with IC_50_ values of 8.7 μM and 6.7 μM, respectively, whereas borrelidin E (**3**) did not display inhibitory activity against any of the tested cancer cell lines. Among the borrelidins, borrelidin (**4**) exhibited the most potent cytotoxicity against all the cell lines that were tested (IC_50_ = 0.8 µM).

## 3. Experimental Section

### 3.1. General Experimental Procedures

Optical rotations were measured with a Jasco P-1020 polarimeter using a 1-cm cell. UV spectra were obtained using a Perkin Elmer Lambda 35 UV/VIS spectrophotometer (Perkin Elmer, Waltham, MA, USA). CD spectra were recorded using an Applied Photophysics Chirascan-Plus circular dichroism spectrometer (Applied Photophysics Ltd., Leatherhead, Surrey, UK). IR spectra were acquired using a Thermo Nicolet iS10 spectrometer (Thermo, Madison, CT, USA). ^1^H, ^13^C, and 2D NMR spectra were obtained using a Bruker Avance 600-MHz (National Center for Interuniversity Research Facilities (NCIRF) at Seoul National University) spectrometer (Bruker, Billerica, MA, USA). Electrospray ionization (ESI) low-resolution LC/MS data were acquired with an Agilent Technologies 6130 quadrupole mass spectrometer coupled with an Agilent Technologies 1200-series HPLC (Agilent Technologies, Santa Clara, CA, USA) using a reversed-phase C_18_ column (Phenomenex Luna, 100 × 4.6 mm) (Phenomenex, Torrence, CA, USA). HR-FAB mass spectra were obtained using a Thermo Scientific Q high-resolution mass spectrometer (Thermo, Madison, CT, USA) at the NCIRF at Seoul National University.

### 3.2. Isolation, Cultivation, and Extraction of the Halophilic Actinomycete Strain 

A saltern topsoil sample was collected on Jeung-do Island in Shinan-gun, Jeollanamdo, Korea. The sample (1 g) was diluted in 10 mL of sterilized artificial saline water (3× the saline concentration of seawater) and vortexed. The mixture was spread onto actinomycete isolation agar, A4 medium (1 L of artificial seawater, 18 g of agar, and 100 mg/L cycloheximide), A5 medium (750 mL of artificial seawater, 250 mL of distilled H_2_O, 18 g of agar, and 100 mg/L cycloheximide), A6 medium (1 L of artificial seawater, 18 g of agar, and 5 mg/L polymyxin B sulfate), A7 medium (1 L of artificial seawater, 18 g of agar, and 5 mg/L kanamycin), and chitin-based agar (1 L of artificial seawater, 4 g of chitin, 0.75 g of K_2_HPO_4_, 0.5 g of MgSO_4_∙7H_2_O, 3.5 g of KH_2_PO_4_, 10 mg of FeSO_4_∙7H_2_O, 10 mg of MnCl_2_∙4H_2_O, 10 mg of ZnSO_4_∙7H_2_O, 18 g of agar, and 100 mg/L cycloheximide). The strain HYJ128 was isolated on actinomycete isolation agar medium. Colonies were repeatedly inoculated onto fresh agar plates to obtain single strains. HYJ128 16S rDNA sequence analysis data obtained from COSMO Co., Ltd. (Cosmogenetech co. Ltd., Seoul, Korea) revealed that HYJ128 is most closely related to *Nocardiopsis lucentensis* (99% identity), identifying the strain as a *Nocardiopsis* sp. (GenBank accession number: LC013479). The HYJ128 strain was cultured in 50 mL of A1+ mannitol medium (4 g of yeast extract, 10 g of starch, 2 g of peptone, and 4 g of mannitol in 1 L of artificial seawater) in a 125-mL Erlenmeyer flask. After the strain was cultivated for five days on a rotary shaker at 200 rpm at 30 °C, 10 mL of the culture was inoculated into 200 mL of YEME medium in a 500-mL Erlenmeyer flask. The strain HYJ128 was further cultivated for five days, and 10 mL of the culture was inoculated into 1 L of the same medium in 2.8-L Fernbach flasks. These large cultures (36 L) were incubated at 200 rpm at 30 °C. After five days, the entire culture volume was extracted twice using ethyl acetate. The ethyl acetate layer was separated and dried over anhydrous sodium sulfate. The ethyl acetate extract was enriched in vacuo to yield 7 g of dried material.

### 3.3. Isolation of the Borrelidins

The dried extract was adsorbed on celite, loaded onto a 20-g C_18_ resin, and fractionated with 200 mL each of 20, 40, 60, 80, and 100% MeOH in H_2_O and 1:1 MeOH/CH_2_Cl_2_. The borrelidins (**1**–**4**) were found in the 60% MeOH/H_2_O fraction. To purify **1**–**4**, the 60% fraction was chromatographed using reversed-phase HPLC with a C_18_ column (Kromasil, 5 μm, 250 × 10 mm) under gradient conditions (45–80% MeOH/water with UV detection at 254 nm and a flow rate of 2 mL/min). Finally, borrelidin C (**1**) (13 mg), borrelidin D (**2**) (7 mg), borrelidin E (**3**) (7 mg), and borrelidin (**4**) (25 mg) eluted as pure compounds at retention times of 45, 47, 31, and 51 min, respectively.

#### 3.3.1. Borrelidin C (**1**)

White powder; ${\left[\alpha \right]}_{D}^{20}$ +20 (*c* 0.1, MeOH); UV (MeOH) *λ*_max_ (log *ε*) 257 (4.50) nm; CD (MeOH) (*Δ ε*) 225 (3.54), 259 (2.70) nm; IR (neat) ν_max_ 3404, 2214, 1717, 1566, 1275 cm^−1^; ^1^H and ^13^C NMR data, see Table 1; HR-FAB-MS *m*/*z* 504.2959 [M − H]^−^ (calcd. for C_28_H_42_NO_7_ 504.2961).

#### 3.3.2. Borrelidin D (**2**)

White powder; ${\left[\alpha \right]}_{D}^{20}$ +15 (*c* 0.1, MeOH); UV (MeOH) *λ*_max_ (log ε) 257 (4.50) nm; CD (MeOH) (*Δ ε*) 223 (3.55), 261 (0.98) nm; IR (neat) ν_max_ 3402, 2216, 1718, 1566, 1265 cm^−1^; ^1^H and ^13^C NMR data, see Table 1; HR-FAB-MS *m*/*z* 504.2958 [M − H]^−^ (calcd. for C_28_H_42_NO_7_ 504.2961).

#### 3.3.3. Borrelidin E (**3**)

White powder; ${\left[\alpha \right]}_{D}^{20}$ +12 (*c* 0.1, MeOH); UV (MeOH) *λ*_max_ (log ε) 257 (4.50) nm; CD (MeOH) (*Δ ε*) 225 (2.23), 255 (2.89) nm; IR (neat) ν_max_ 3444, 2204, 1710, 1555, 1273 cm^−1^; ^1^H and ^13^C NMR data, see Table 1; HR-FAB-MS *m*/*z* 504.2966 [M − H]^−^ (calcd. for C_28_H_42_NO_7_ 504.2961).

### 3.4. MTPA Esterification of Borrelidins C–D (**1**–**2**)

Each compound (1 mg) was prepared in two 40-mL vials and dried for eight hours in vacuo. A volume of 1 mL of anhydrous pyridine was injected under argon gas into each vial to dissolve the compound. Each vial was stirred for 15 min at room temperature. After 15 min, *R*-MTPA chloride and *S*-MTPA chloride (20 μL) were added separately to the reaction vials. The reactions were terminated after 4 h by adding MeOH. The esterified products were purified via HPLC using a C_18_ column (Kromasil, 5 μm, 250 × 10 mm) with a gradient of 50% MeOH/water to 100% MeOH over 45 min, at a flow rate of 2 mL/min, and using UV absorbance at 254 nm for detection. Bis-MTPA esters (**1a**, **1b**, **2a**, and **2b**) eluted at the retention times of 50, 51, 52, and 54 min, respectively. ^1^H NMR, COSY, and TOCSY NMR experiments were performed to calculate *Δδ_S-R_* values.

#### 3.4.1. Bis-*S*-MTPA Ester (**1a**) of Borrelidin C (**1**)

^1^H NMR (600 MHz, pyridine-*d*_5_) *δ* 7.78–7.72 (m, 4H), 7.50–7.42 (m, 6H), 7.06–6.99 (m, 2H), 6.40 (m, 1H), 5.75 (d, *J* = 11.0, 1H), 5.73 (d, *J* = 11.0, 1H), 5.31 (m, 1H), 5.06 (m, 1H), 3.62 (s, 3H), 3.60 (s, 3H), 2.90 (m, 1H), 2.85 (s, 1H), 2.83 (m, 1H), 2.8 (m, 1H), 2.66 (m, 1H), 2.62 (m, 1H), 2.57 (m, 1H), 2.56 (m, 1H), 2.42 (m, 1H), 2.33 (m, 1H), 2.16 (dd, *J* = 14.0, 7.0, 1H), 1.72–1.62 (m, 3H), 1.61–1.53 (m, 3H), 1.42 (m, 1H), 1.23–1.13 (m, 2H), 1.03 (d, *J* = 6.0, 3H), 1.01 (d, *J* = 6.5, 3H), 0.95 (d, *J* = 6.0, 3H), 0.92 (d, *J* = 5.5, 3H).

#### 3.4.2. Bis-*R*-MTPA Ester (**1b**) of Borrelidin C (**1**)

^1^H NMR (600 MHz, pyridine-*d*_5_) *δ* 7.80–7.73 (m, 4H), 7.51–7.41 (m, 6H), 7.08–7.01 (m, 2H), 6.38 (m, 1H), 5.74 (d, *J* = 11.0, 1H), 5.70 (m, 1H), 5.40 (m, 1H), 5.01 (m, 1H), 3.78 (s, 3H), 3.62 (s, 3H), 2.87–2.78 (m, 4H), 2.67 (m, 1H), 2.65 (m, 1H), 2.57 (m, 1H), 2.56 (m, 1H), 2.39 (m, 1H), 2.30 (m, 1H), 2.06 (dd, *J* = 14.0, 7.0, 1H), 1.74–1.64 (m, 2H), 1.63–1.56 (m, 3H), 1.54 (m, 1H), 1.40 (t, *J* = 12.5, 1H), 1.20–1.14 (m, 2H), 1.01 (d, *J* = 7.0, 3H), 0.92 (d, *J* = 6.0, 3H), 0.90 (d, *J* = 6.0, 3H), 0.74 (m, 3H).

#### 3.4.3. Bis-*S*-MTPA Ester (**2a**) of Borrelidin D (**2**)

^1^H NMR (600 MHz, pyridine-*d*_5_) *δ* 7.85–7.82 (m, 2H), 7.79–7.76 (m, 2H), 7.57–7.44 (m, 6H), 7.23 (m, 1H), 6.85 (dd, *J* = 14.0, 11.0, 1H), 6.43 (ddd, *J* = 14.0, 11.0, 5.0, 1H), 5.91 (d, *J* = 11.0, 1H), 5.73 (d, *J* = 11.0, 1H), 5.23 (m, 1H), 4.93 (m, 1H), 3.74 (s, 3H), 3.66 (s, 3H), 3.18 (dd, *J* = 17.0, 2.0, 1H), 2.69 (dd, *J* = 17.0, 11.0, 1H), 2.62 (m, 1H), 2.56–2.54 (m, 2H), 2.40 (m, 1H), 2.31 (m, 1H), 2.13 (m, 1H), 1.98–1.94 (m, 2H), 1.87 (d, *J* = 11.0, 1H), 1.67 (m, 1H), 1.56 (dd, *J* = 13.0, 3.0, 1H), 1.42 (m, 1H), 1.30–1.25 (m, 2H), 1.13–1.08 (m, *2*H), 1.07 (d, *J* = 6.0, 3H), 1.04 (dd, *J* = 8.5, 2.0, 1H), 1.02 (d, *J* = 1.5, 1H), 0.97 (d, *J* = 7.0, 3H), 0.90 (d, *J* = 6.5, 3H), 0.87 (d, *J* = 6.0, 3H).

#### 3.4.4. Bis-*R*-MTPA Ester (**2b**) of Borrelidin D (**2**)

^1^H NMR (600 MHz, pyridine-*d*_5_) *δ* 7.93–7.89 (m, 2H), 7.85–7.82 (m, 2H), 7.54–7.46 (m, 6H), 7.26 (m, 1H), 6.90 (dd, *J* = 15.0, 11.0, 1H), 6.44 (m, 1H), 5.81 (d, *J* = 11.0, 1H), 5.76 (d, *J* = 11.0, 1H), 5.27 (m, 1H), 4.91 (m, 1H), 3.78 (s, 3H), 3.76 (s, 3H), 3.21 (dd, *J* = 17.5, 2.0, 1H), 2.75 (dd, *J* = 17.5, 11.0, 1H), 2.66 (m, 1H), 2.53–2.48 (m, 2H), 2.41 (m, 1H), 2.31 (m, 1H), 2.12 (m, 1H), 1.97 (d, *J* = 11.0, 1H), 1.94–1.85 (m, 2H), 1.65 (m, 1H), 1.56 (dd, *J* = 13.0, 3.0, 1H), 1.43 (m, 1H), 1.25–1.19 (m, 2H), 1.08–1.00 (m, 4H), 0.95 (d, *J* = 7.0, 3H), 0.89 (d, *J* = 7.0, 3H), 0.88 (d, *J* = 7.0, 3H), 0.74 (d, *J* = 6.5, 3H).

### 3.5. Antibacterial Activity Assay

Gram-positive bacteria (*S. aureus* ATCC 25923, *E. faecalis* ATCC 19433, *E. faecium* ATCC 19434) and Gram-negative bacteria (*P. hauseri* NRBC 3851, *K. pneumoniae* ATCC 10031, *S. enterica* ATCC 14028, *E. coli* ATCC 25922) were used for antimicrobial activity tests. Bacteria were grown overnight in Luria Bertani (LB) broth (Becton, Dickinson and Company, Franklin Lakes, NJ, USA) at 37 °C, harvested by centrifugation, and then washed twice with sterile distilled water. Stock solutions of the borrelidins (**1**–**4**) were prepared in DMSO. Each stock solution was diluted with m Plate Count Broth (Difco) to prepare serial two-fold dilutions in the range of 50 to 0.8 μg/mL. Ten microliters of the broth, containing approximately 10^5^ colony-forming units (cfu)/mL of test bacteria, was added to each well of a 96-well microliter plate. The culture plates were incubated for 12 h at 37 °C. The MIC values were determined as the lowest concentration of test compound that inhibited bacterial growth. Ampicillin was used as a reference compound.

### 3.6. Cytotoxicity Assay

The effect of borrelidins (**1**–**4**) on cell proliferation was measured by the sulforhodamine B (SRB) cellular protein-staining method with some modifications. In brief, A549 (lung cancer), HCT116 (colon cancer), SNU638 (stomach cancer), SK-HEP1 (liver cancer), MDA-MB231 (breast cancer), and K562 (leukemia) cells (1 × 10^4^ cells in 190 μL of complete DMEM) were seeded in 96-well plates with various concentrations of borrelidins (**1**–**4**) and incubated at 37 °C in a humidified atmosphere with 5% CO_2_. After 72 h of borrelidin (**1**–**4**) treatment, the cells were fixed with 10% TCA solution for 1 h, and cellular proteins were stained with a solution of 0.4% SRB in 1% acetic acid. The stained cells were dissolved in 10 mM Tris buffer (pH 10.0). The effect of borrelidins (**1**–**4**) on cell viability was calculated as a percentage relative to a solvent-treated control, and the IC_50_ values were calculated using a nonlinear regression analysis (percent survival versus concentration). Etoposide was used as a positive control.

## 4. Conclusions

Chemical investigation of the rare saltern-derived actinomycete *Nocardiopsis* sp. strain HYJ128 resulted in the discovery of new members of the borrelidin class of antibiotics. Borrelidins C and D (**1**, **2**) and borrelidin (**4**) displayed antibacterial inhibitory activity, particularly against the Gram-negative pathogen *S. enterica*, indicating that the borrelidin class could be a useful scaffold to develop antibiotics against *S. enterica*. Moreover, additional hydroxylation at C-20 or C-7 of borrelidin, resulting in borrelidins C–E, significantly reduced or even abolished antibacterial activity. Borrelidins C–D exhibited significant cytotoxic effects against the SNU638 and K562 carcinoma cell lines, whereas borrelidin E did not show any significant activity in these assays. Borrelidins C–E are rare members of the borrelidin class of antibiotics. Although borrelidin was initially reported in a terrestrial *Streptomyces* sp. in the early days of antibiotic discovery in 1949 [9], only one additional member, borrelidin B, in which the nitrile functional group was reduced to a primary amine, has been reported, and this member was isolated from a marine sediment-derived *Streptomyces* sp. in 2014 [14]. The discovery of borrelidins C–E as the first secondary metabolites from a saltern-derived rare actinomycete genus *Nocardiposis* emphasizes the unexplored potential of halophilic rare actinomycetes inhabiting extremely saline saltern environments.

## Figures and Tables

**Table 1:** NMR data for **1**–**3** in pyridine-*d*_5_.

C/H	1			2			3		
	*δ*_H_^a^	Mult (*J* in Hz)	*δ*_C_^b^	*δ*_H_^a^	Mult (*J* in Hz)	*δ*_C_^b^	*δ*_H_^a^	Mult (*J* in Hz)	*δ*_C_^b^
1	–	–	172.4 s	–	–	172.7 s	–	–	172.7 s
2	2.79	m	39.3 t	2.82	m	41.1 t	2.92	m	41.3 t
	2.76	m		2.80	m		2.79	m	
3	4.37	m	70.8 d	4.34	m	71.2 d	4.44	m	69.8 d
4	1.98	m	36.5 d	1.92	m	36.9 d	1.96	m	36.8 d
5	1.34	m	43.9 t	1.26	m	44.9 t	1.72	m	41.5 t
	0.95	m		0.94	m		1.66	m	
6	2.10	m	27.6 d	2.00	m	27.9 d	2.37	m	32.2 d
7	1.07	m	48.1 t	1.05	m	48.6 t	3.26	br d (9.5)	81.5 d
	1.00	m		0.95	m				
8	1.73	m	26.8 d	1.72	m	27.1 d	2.01	m	34.4 d
9	1.41	ddd (13.0, 13.0, 2.5)	37.9 t	1.38	ddd (13.0, 13.0, 2.5)	38.1 t	1.37	m	36.3 t
	0.98	m		0.99	m		1.15	m	
10	2.28	m	35.6 d	2.30	m	36.0 d	2.36	m	36.1 d
11	4.57	d (9.5)	72.2 d	4.58	d (9.5)	72.6 d	4.62	d (9.5)	72.7 d
12	–	–	120.1 s	–	–	120.6 s	–	–	120.6 s
13	6.83	d (11.0)	143.7 d	6.76	d (11.5)	143.2 d	6.83	d (11.0)	143.4 d
14	6.66	dd (14.0, 11.0)	127.7 d	6.68	dd (14.0, 11.5)	128.1 d	6.71	dd (14.0, 11.0)	127.9 d
15	6.33	m	139.1 d	6.27	ddd (14.0, 11.0, 4.0)	139.5 d	6.33	ddd (14.0, 11.0, 4.0)	139.9 d
16	2.60	m	36.2 t	2.57	m	36.6 t	2.57	m	37.0 t
	2.57	m		2.49	m		2.5	m	
17	5.65	m	76.7 d	5.37	m	76.8 d	5.38	m	76.8 d
18	3.22	m	44.7 d	3.49	m	45.1 d	3.09	m	46.8 d
19	2.42	m	39.1 t	2.26	m	40.8 t	1.90	m	30.3 t
	1.80	m		1.60	m		1.27	m	
20	4.79	m	72.4 d	4.59	m	73.3 d	1.79	m	26.2 t
							1.62	m	
21	2.46	m	41.6 t	2.38	m	40.8 t	2.14	m	32.3 t
	2.41	m		2.20	m		1.98	m	
22	3.44	ddd (8.5, 8.5, 8.5)	48.3 d	3.07	ddd (8.0, 8.0, 8.0)	49.7 d	2.89	ddd (7.5, 7.5, 7.5)	50.8 d
23	–	–	179.4 s	–	–	181.9 s	–	–	181.2 s
24	0.85	d (7.0)	18.3 q	0.89	d (7.0)	18.9 q	0.92	d (7.0)	17.9 q
25	0.97	d (6.5)	18.6 q	0.92	d (6.5)	18.7 q	1.18	d (6.5)	11.6 q
26	0.91	d (6.5)	20.6 q	0.90	d (6.5)	21.0 q	1.31	d (6.5)	16.7 q
27	1.28	d (6.5)	15.4 q	1.30	d (6.5)	15.7 q	1.37	d (6.5)	15.8 q
28	–	–	118.3 s	–	–	118.6 s	–	–	118.6 s

^a^ 600 MHz; ^b^ 150 MHz.

**Table 2:** Antibacterial activities of the borrelidins (**1**–**4**) against pathogenic bacteria.

MIC in µM	Gram-Positive			Gram-Negative			
	*S. aureus*	*E. faecalis*	*E. faecium*	*P. hauseri*	*K. pneumoniae*	*S. enterica*	*E. coli*
Borrelidin C (**1**)	>250	250	>250	>250	>250	16	>250
Borrelidin D (**2**)	>250	>250	>250	>250	>250	63	>250
Borrelidin E (**3**)	250	>250	>250	>250	>250	250	>250
Borrelidin (**4**)	>260	33	65	16	65	0.51	260
Ampicillin	0.37	5.7	5.7	0.37	>367	1.4	23

**Table 3:** Cytotoxicities of the borrelidins (**1**–**4**) against cancer cell lines.

IC_50_ in µM	A549	HCT116	SNU638	SK-HEP1	MDA-MB231	K562
Borrelidin C (**1**)	9.1	10	5.5	63	96	5.7
Borrelidin D (**2**)	12	15	8.7	71	64	6.7
Borrelidin E (**3**)	>100	>100	>100	>100	>100	>100
Borrelidin (**4**)	0.8	0.8	0.8	0.8	0.8	0.8
Etoposide	0.68	14	0.57	8.7	5.4	1.5

## Supplementary Materials

The following are available online at www.mdpi.com/1660-3397/15/6/166/s1. Figures S1–S6: ^1^H, ^13^C, COSY, HSQC, HMBC, and ROESY NMR data of **1** in pyridine-*d*_5_, Figures S7–S12: ^1^H, ^13^C, COSY, HSQC, HMBC, and ROESY NMR data of **2** in pyridine-*d*_5_, Figures S13–S18: ^1^H, ^13^C, COSY, HSQC, HMBC, and ROESY NMR data of **3** in pyridine-*d*_5_, Figures S19 and S20: ^1^H and COSY NMR data of S-MTPA ester (**1a**) of **1** in pyridine-*d*_5_, Figures S21 and S22: ^1^H and COSY NMR data of R-MTPA ester (**1b**) of **1** in pyridine-*d*_5_, Figures S23 and S24: ^1^H and COSY NMR data of S-MTPA ester (**2a**) of **2** in pyridine-*d*_5_, Figures S25 and S26: ^1^H and COSY NMR data of S-MTPA ester (**2b**) of **2** in pyridine-*d*_5_, Figure S27: ^1^H NMR data of homo-decoupling experiment by irradiation ^1^H at *δ* 2.38 for borrelidin E (**3**) in pyridine-*d*_5_, Figure S28: Experimental CD spectra of **1**–**4**.
