# Supplementary material for: Borrelidins C–E: New Antibacterial Macrolides from a Saltern-Derived Halophilic Nocardiopsis sp
Source: Mar Drugs. 2017 Jun 6;15(6):166. doi: 10.3390/md15060166 (PMC5484116; doi:10.3390/md15060166)

Supplementary Material:

Borrelidins C-E: New Antibacterial Macrolides from a Saltern-Derived Halophilic *Nocardiopsis* sp.

Jungwoo Kim[1], Daniel Shin[1], Seong-Hwan Kim[1], Wanki Park[2], Yoonho Shin[1], Won Kyung Kim[1], Sang Kook Lee[1], Ki-Bong Oh[2], Jongheon Shin[1], and Dong-Chan Oh[1,*]

[1] Natural Products Research Institute, College of Pharmacy, Seoul National University, Seoul 08826, Republic of Korea; E-Mail: xyrho25@gmail.com (J.K.); pharmcraft87@snu.ac.kr (D.S.); yanberk@snu.ac.kr (S.H.K.); adamas2001@hanmail.net (W.P); dicafree@korea.com (Y.S.); pooh6764@snu.ac.kr (W.K.K); sklee61@snu.ac.kr (S.K.L.); shinj@snu.ac.kr (J.S.)

[2] Department of Agricultural Biotechnology, College of Agriculture and Life Sciences, Seoul National University, Seoul 08826, Republic of Korea; E-Mail: ohkibong@snu.ac.kr (K.-B.O.)

* Correspondence: dongchanoh@snu.ac.kr; Tel.: +82-2-880-2491; Fax: +82-2-762-8322.

Table of Contents

Figure S1. ^{1}H NMR spectrum (600 MHz) of borrelidin C (**1**) in pyridine-d_5.
Figure S2. ^{13}C NMR spectrum (150 MHz) of borrelidin C (**1**) in pyridine-d_5.
Figure S3. COSY NMR spectrum of borrelidin C (**1**) in pyridine-d_5.
Figure S4. HSQC NMR spectrum of borrelidin C (**1**) in pyridine-d_5.
Figure S5. HMBC NMR spectrum of borrelidin C (**1**) in pyridine-d_5.
Figure S6. ROESY NMR spectrum of borrelidin C (**1**) in pyridine-d_5.
Figure S7. ^{1}H NMR spectrum (600 MHz) of borrelidin D (**2**) in pyridine-d_5.
Figure S8. ^{13}C NMR spectrum (150 MHz) of borrelidin D (**2**) in pyridine-d_5.
Figure S9. COSY NMR spectrum of borrelidin D (**2**) in pyridine-d_5.
Figure S10. HSQC NMR spectrum of borrelidin D (**2**) in pyridine-d_5.
Figure S11. HMBC NMR spectrum of borrelidin D (**2**) in pyridine-d_5.
Figure S12. ROESY NMR spectrum of borrelidin D (**2**) in pyridine-d_5.
Figure S13. ^{1}H NMR spectrum (600 MHz) of borrelidin E (**3**) in pyridine-d_5.
Figure S14. ^{13}C NMR spectrum (150 MHz) of borrelidin E (**3**) in pyridine-d_5.
Figure S15. COSY NMR spectrum of borrelidin E (**3**) in pyridine-d_5.
Figure S16. HSQC NMR spectrum of borrelidin E (**3**) in pyridine-d_5.
Figure S17. HMBC NMR spectrum of borrelidin E (**3**) in pyridine-d_5.
Figure S18. ROESY NMR spectrum of borrelidin E (**3**) in pyridine-d_5.
Figure S19. ^{1}H NMR spectrum of bis-*S*-MTPA ester of borrelidin C (**1a**) at 600 MHz in pyridine-d_5.
Figure S20. COSY NMR spectrum of bis-*S*-MTPA ester of borrelidin C (**1a**) at 600 MHz in pyridine-d_5.
Figure S21. ^{1}H NMR spectrum of bis-*R*-MTPA ester of borrelidin C (**1b**) at 600 MHz in pyridine-d_5
Figure S22. COSY NMR spectrum of bis-*R*-MTPA ester of borrelidin C (**1b**) at 600 bis-MHz in pyridine-d_5.
Figure S23. ^{1}H NMR spectrum of bis-*S*-MTPA ester of borrelidin D (**2a**) at 600 bis-MHz in pyridine-d_5.
Figure S24. COSY NMR spectrum of bis-*S*-MTPA ester of borrelidin D (**2a**) at 600 MHz in pyridine-d_5.
Figure S25. ^{1}H NMR spectrum of bis-*R*-MTPA ester of borrelidin D (**2b**) at 600 MHz in pyridine-d_5.
Figure S26. COSY NMR spectrum of bis-*R*-MTPA ester of borrelidin D (**2b**) at 600 MHz in pyridine-d_5.
Figure S27. ^{1}H NMR spectrum of homo-decoupling ^{1}H experiment by irradiation ^{1}H at δ 2.38 of borrelidin E (**3**) at 600 MHz in pyridine-d_5.
Figure S28. Experimental CD spectra of **1-4**.

Figure S1. ^{1}H NMR spectrum (600 MHz) of borrelidin C (**1**) in pyridine-d_5.

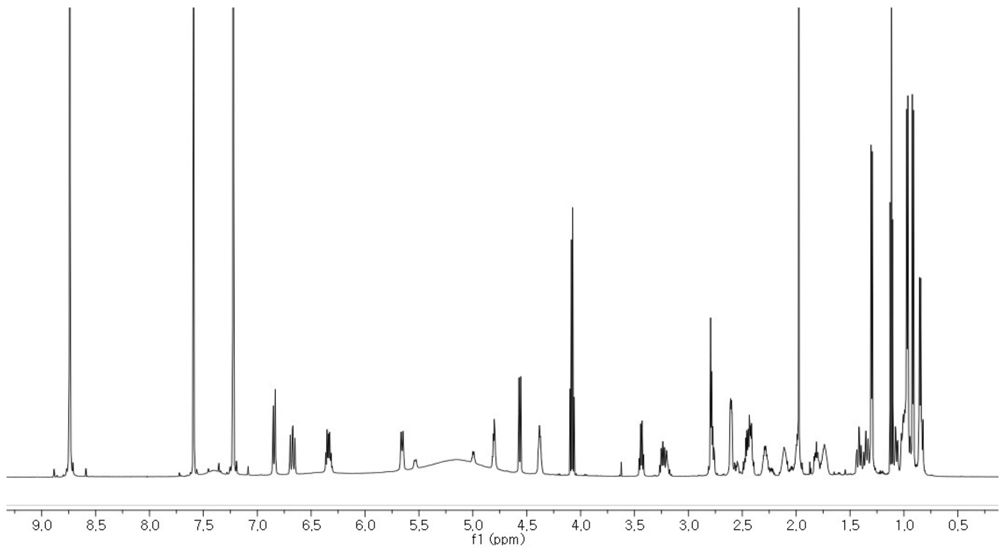

Figure S2. ^{13}C NMR spectrum (150 MHz) of borrelidin C (**1**) in pyridine-d_5.

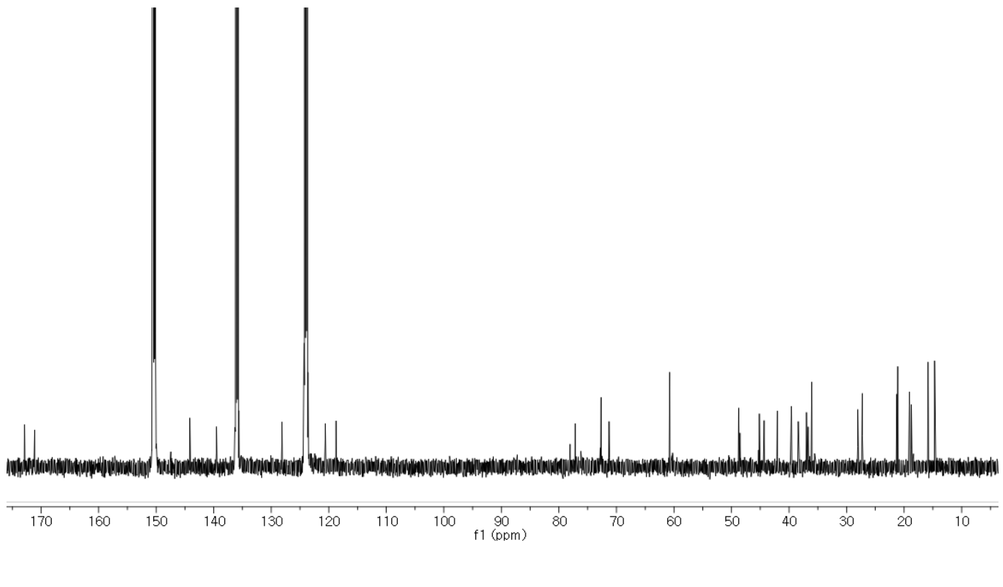

Figure S3. COSY NMR spectrum of borrelidin C (**1**) in pyridine-d_5.

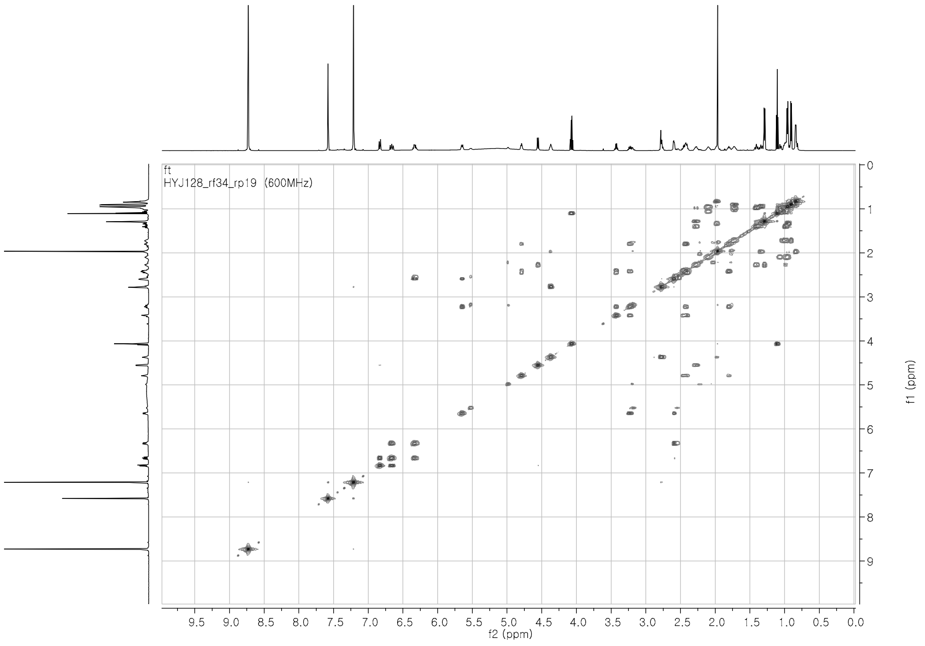

Figure S4. HSQC NMR spectrum of borrelidin C (**1**) in pyridine-d_5.

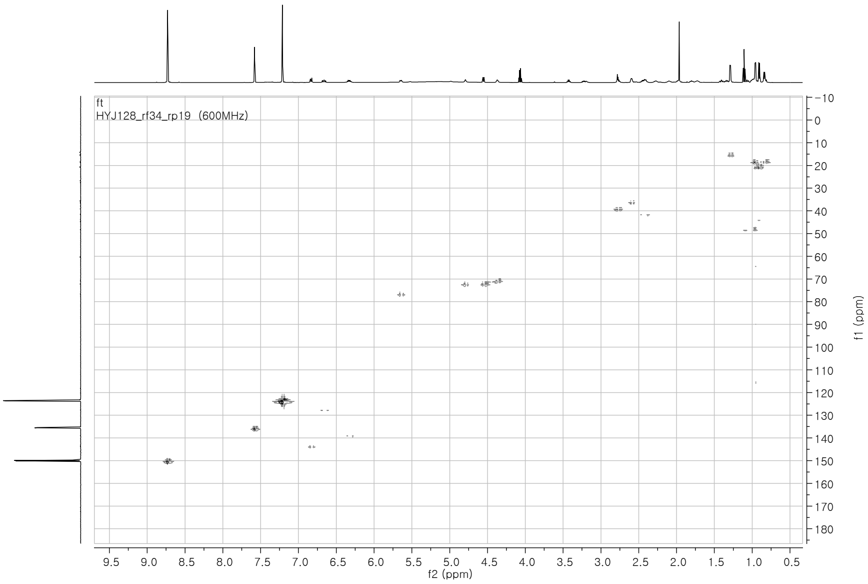

Figure S5. HMBC NMR spectrum of borrelidin C (**1**) in pyridine-d_5.

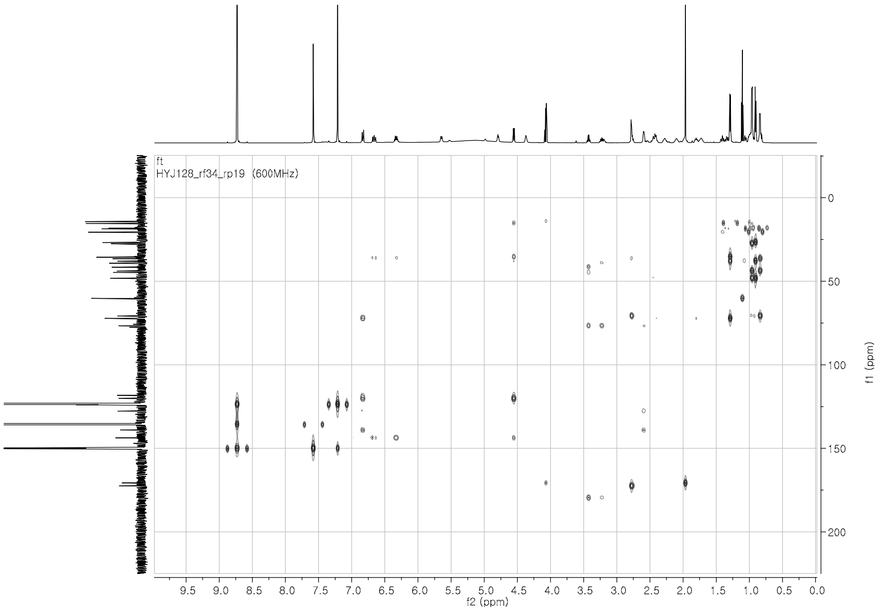

Figure S6. ROESY NMR spectrum of borrelidin C (**1**) in pyridine-d_5.

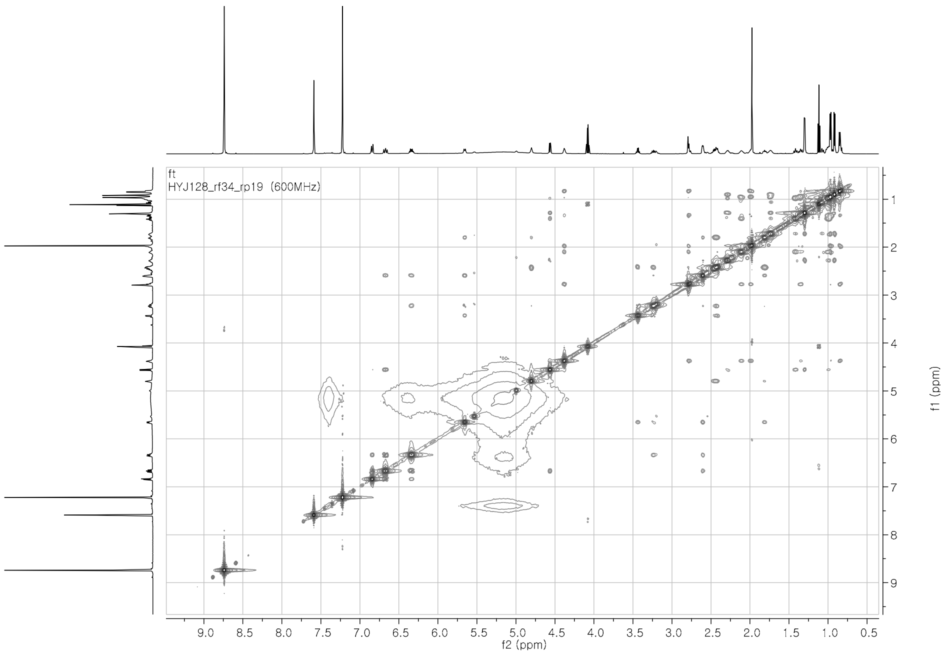

Figure S7. ^{1}H NMR spectrum (600 MHz) of borrelidin D (**2**) in pyridine-d_5.

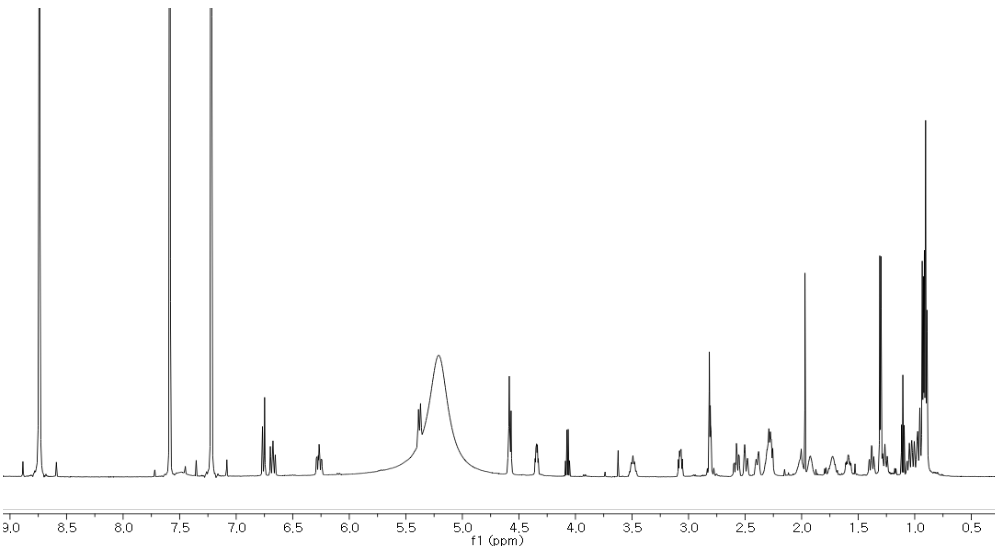

Figure S8. ^{13}C NMR spectrum (150 MHz) of borrelidin D (**2**) in pyridine-d_5.

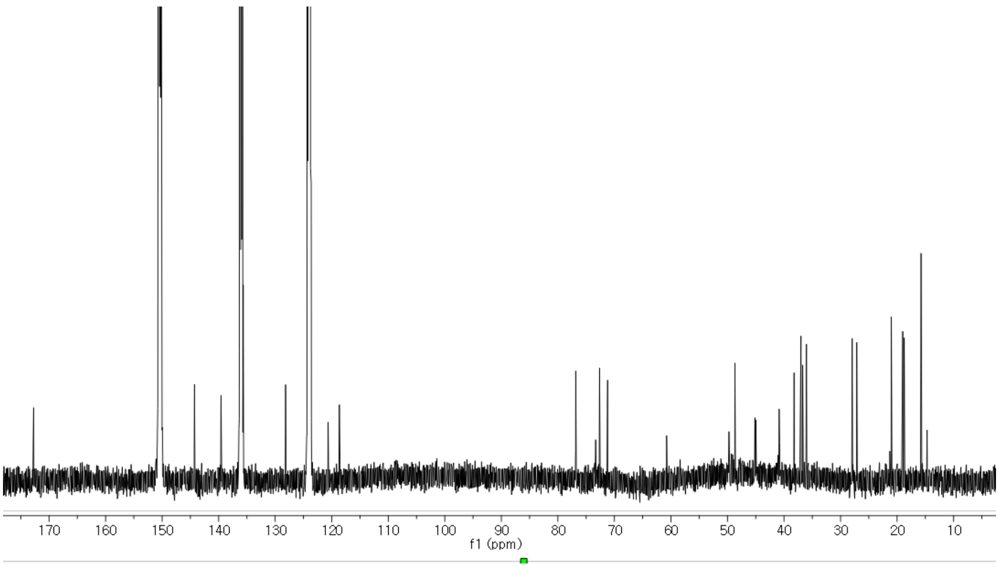

Figure S9. COSY NMR spectrum of borrelidin D (**2**) in pyridine-d_5.

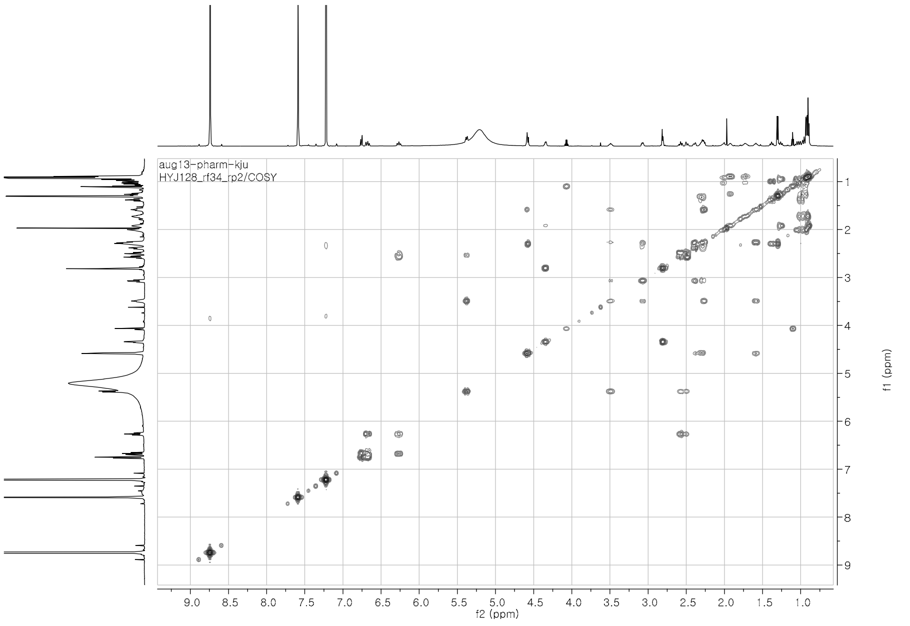

Figure S10. HSQC NMR spectrum of borrelidin D (**2**) in pyridine-d_5.

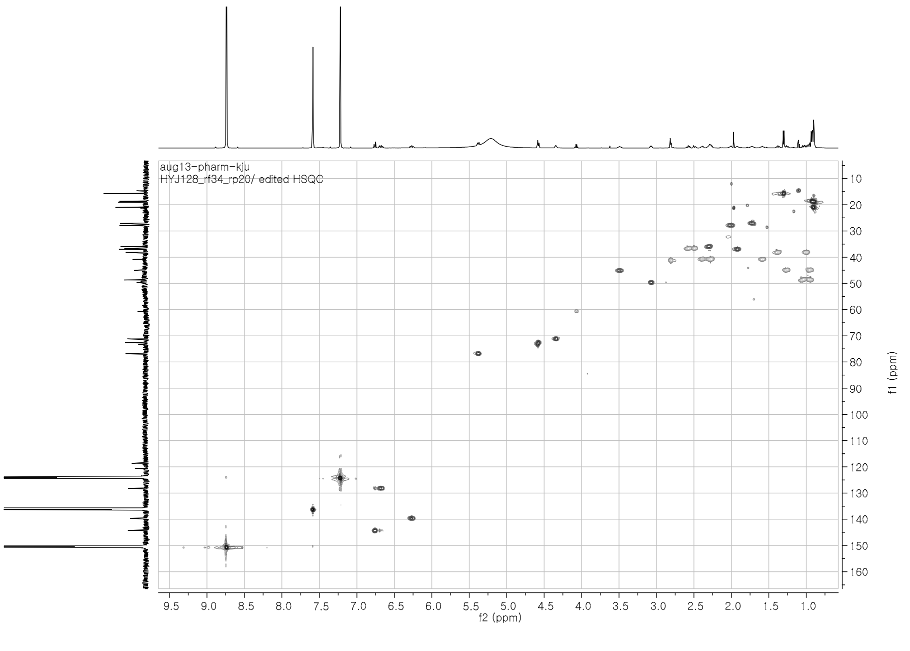

Figure S11. HMBC NMR spectrum of borrelidin D (**2**) in pyridine-d_5.

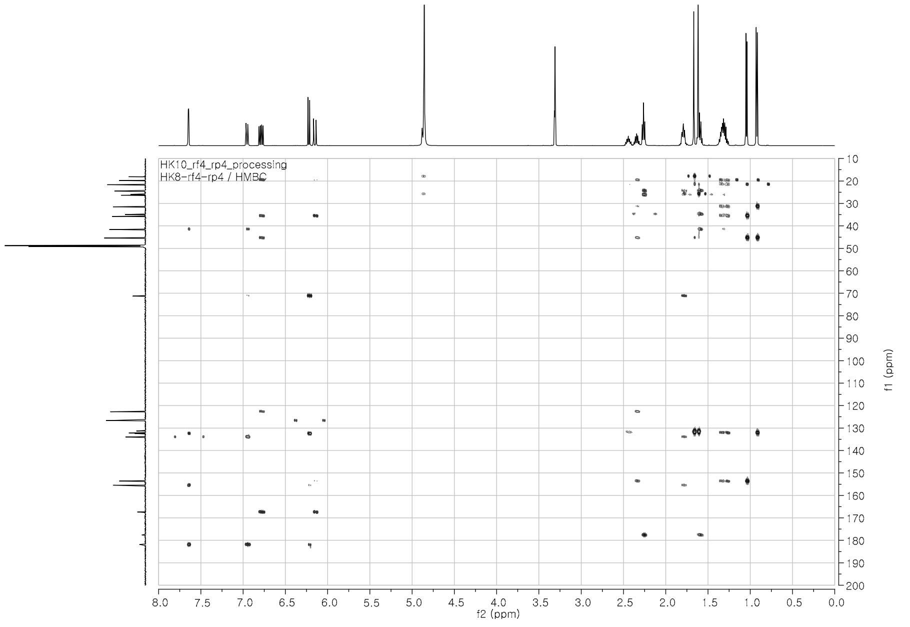

Figure S12. ROESY NMR spectrum of borrelidin D (**2**) in pyridine-d_5.

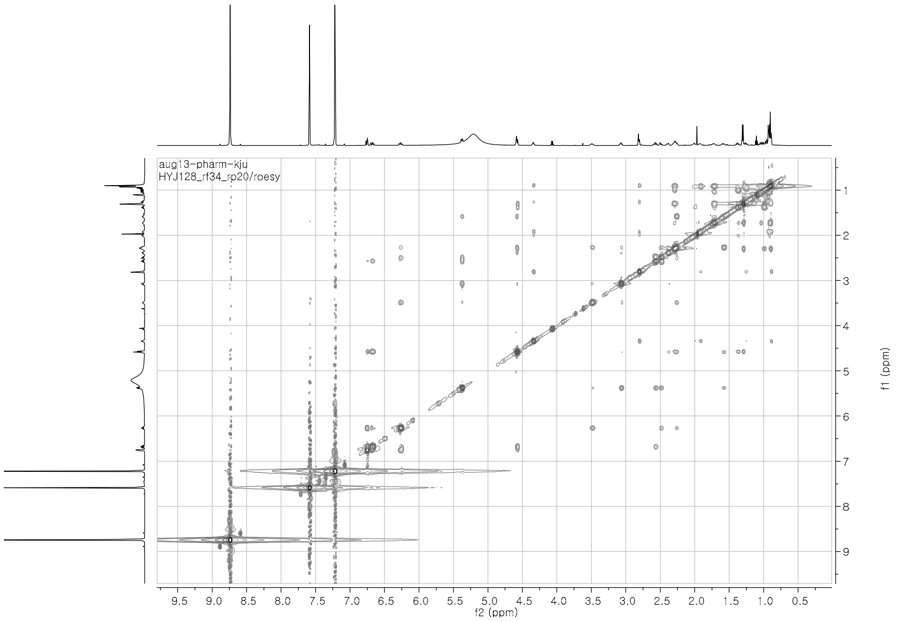

Figure S13. ^{1}H NMR spectrum (600 MHz) of borrelidin E (**3**) in pyridine-*d*5.

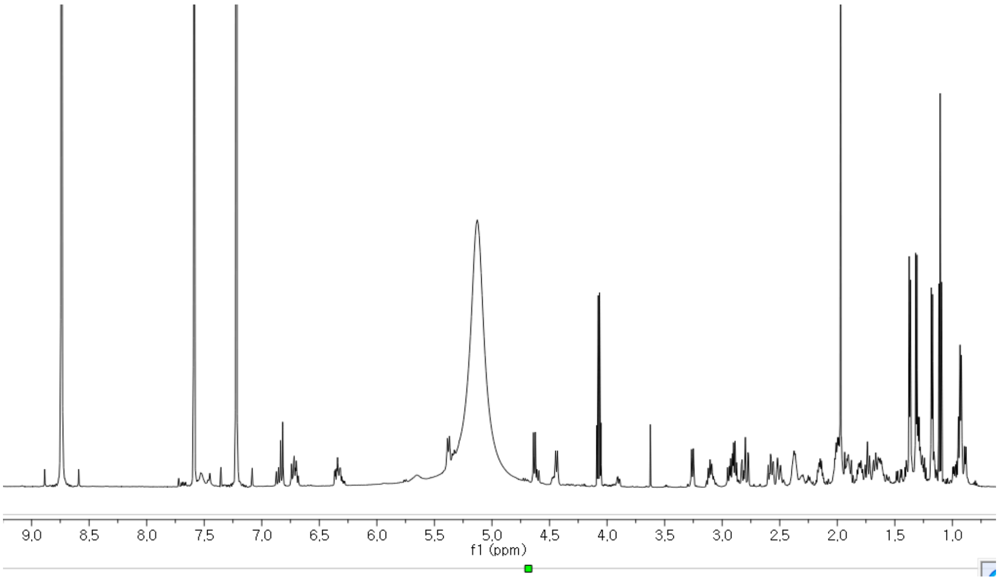

Figure S14. ^{13}C NMR spectrum (150 MHz) of borrelidin E (**3**) in pyridine-*d*5.

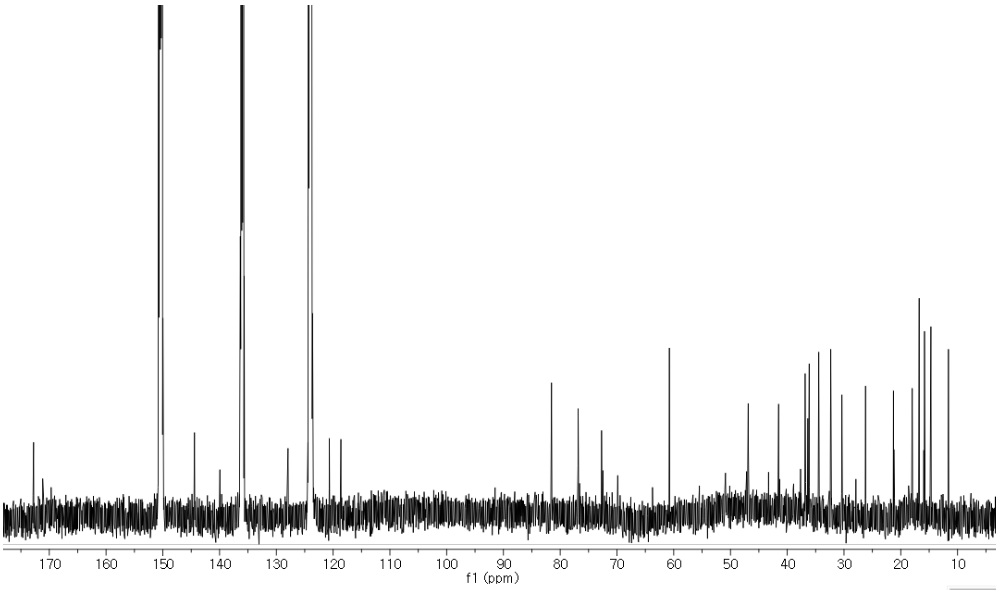

Figure S15. COSY NMR spectrum of borrelidin E (**3**) in pyridine-d_5.

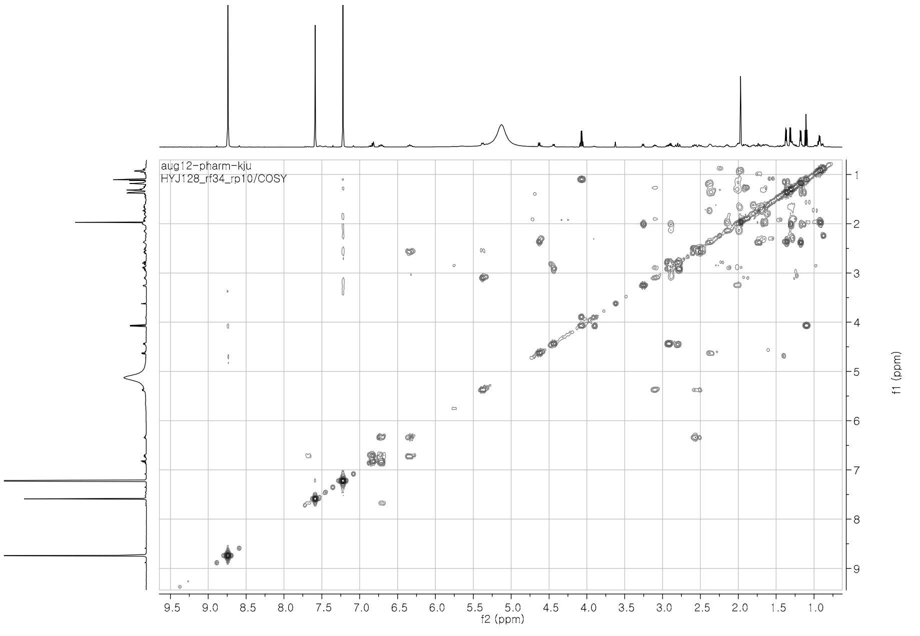

Figure S16. HSQC NMR spectrum of borrelidin E (**3**) in pyridine-d_5.

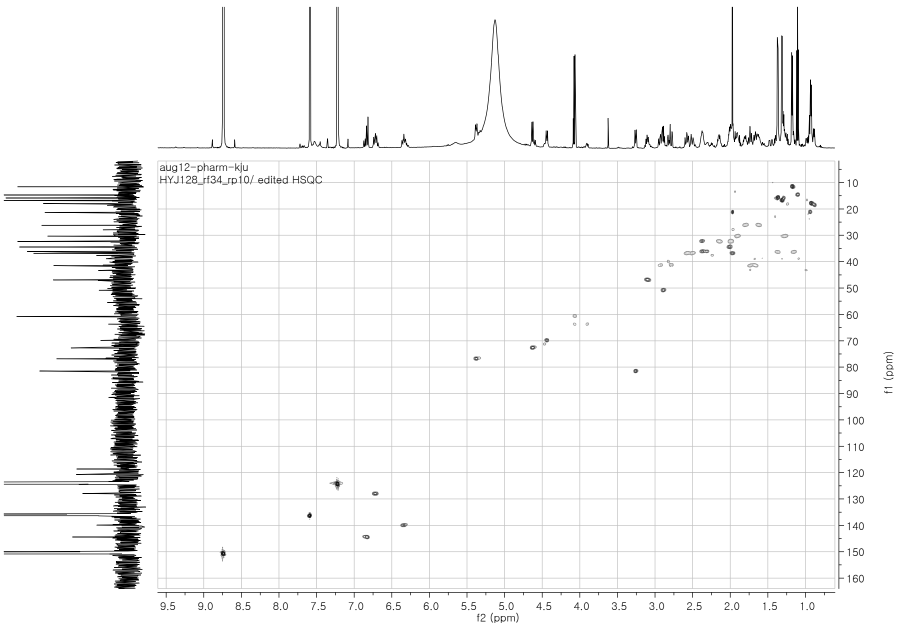

Figure S17. HMBC NMR spectrum of borrelidin E (**3**) in pyridine-*d*5.

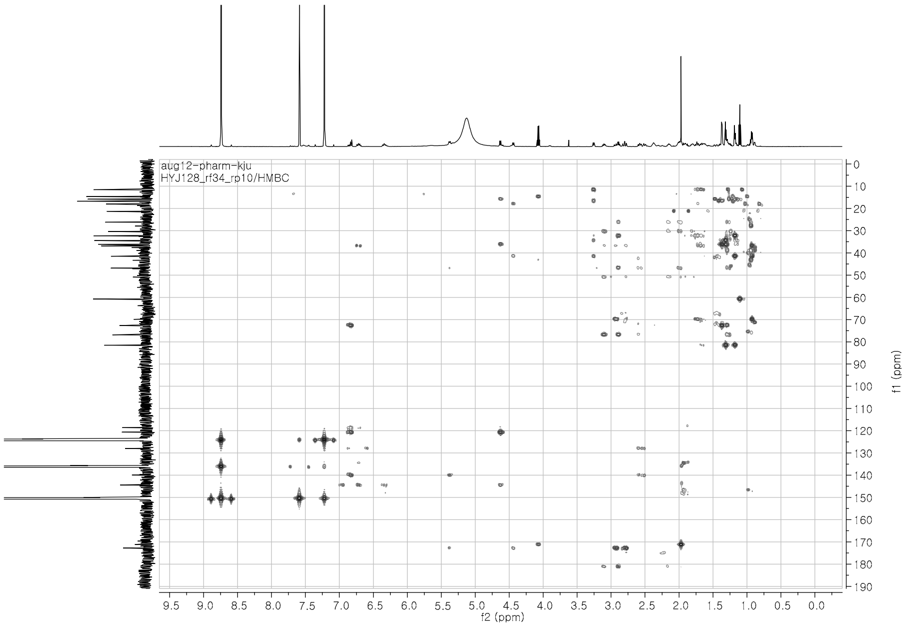

Figure S18. ROESY NMR spectrum of borrelidin E (**3**) in pyridine-*d*5.

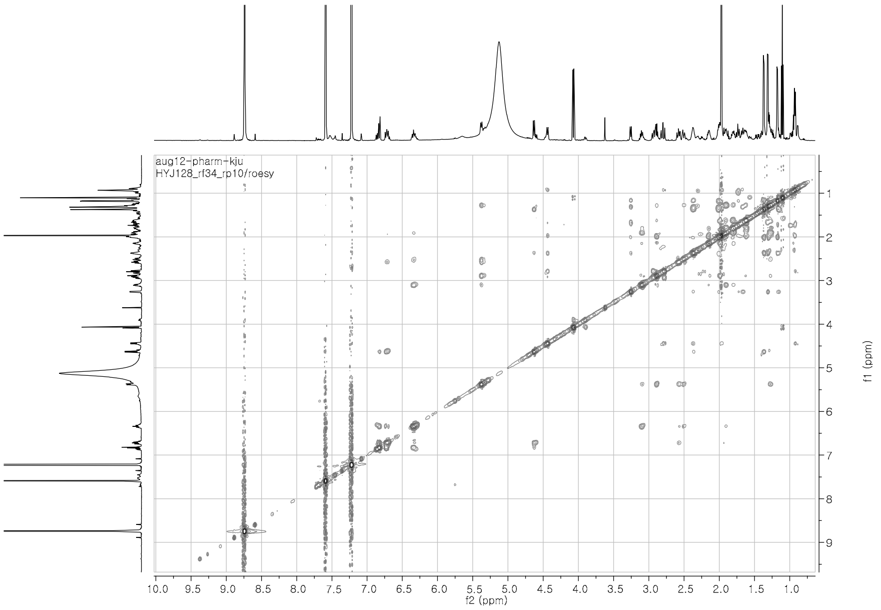

Figure S19. ^{1}H NMR spectrum of bis-*S*-MTPA ester of borrelidin C (**1a**) at 600 MHz in pyridine-d_5.

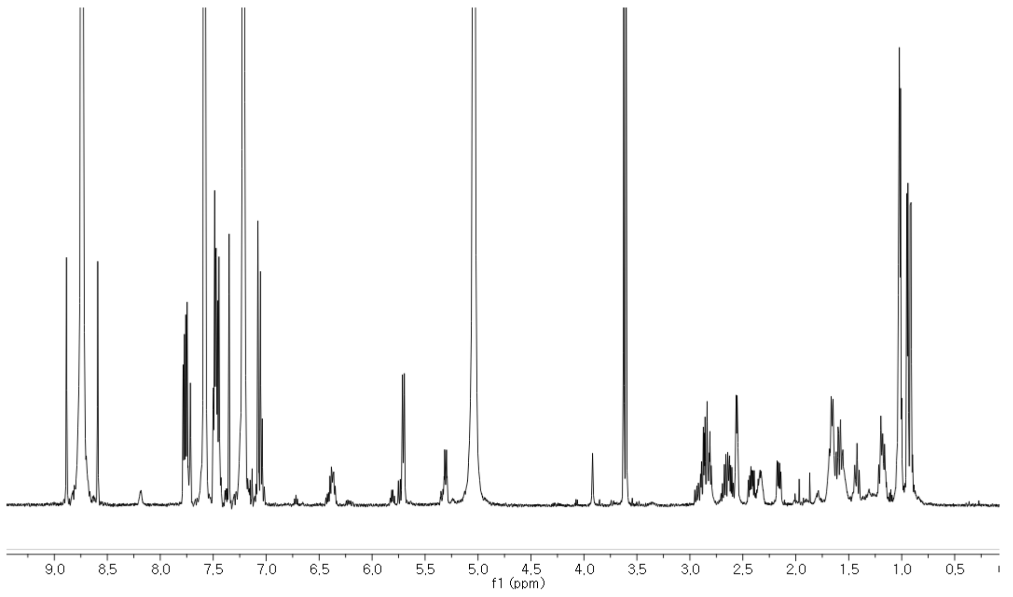

Figure S20. COSY NMR spectrum of bis-*S*-MTPA ester of borrelidin C (**1a**) at 600 MHz in pyridine-d_5.

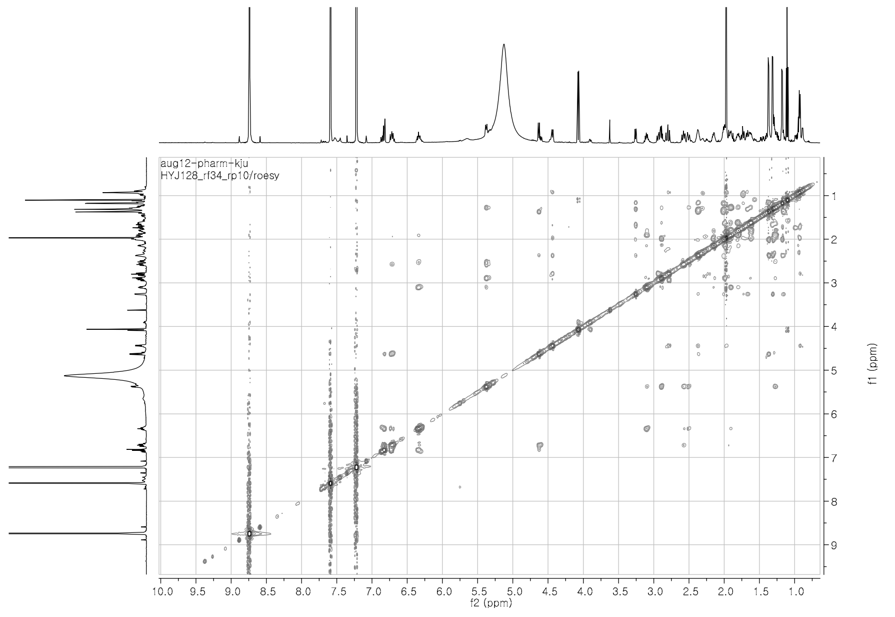

Figure S21. ^{1}H NMR spectrum of bis-*R*-MTPA ester of borrelidin C (**1b**) at 600 MHz in pyridine-d_5.

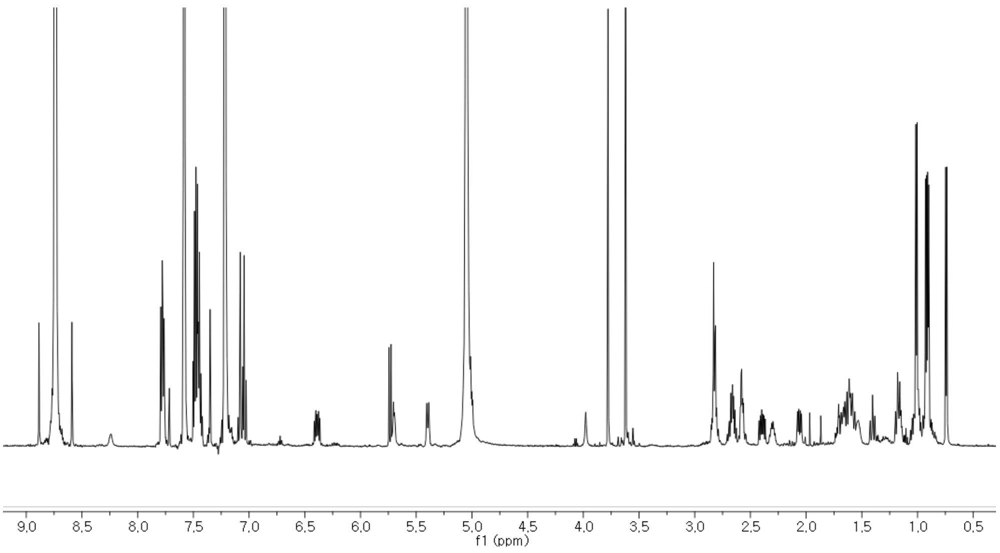

Figure S22. COSY NMR spectrum of bis-*R*-MTPA ester of borrelidin C (**1b**) at 600 MHz in pyridine-d_5.

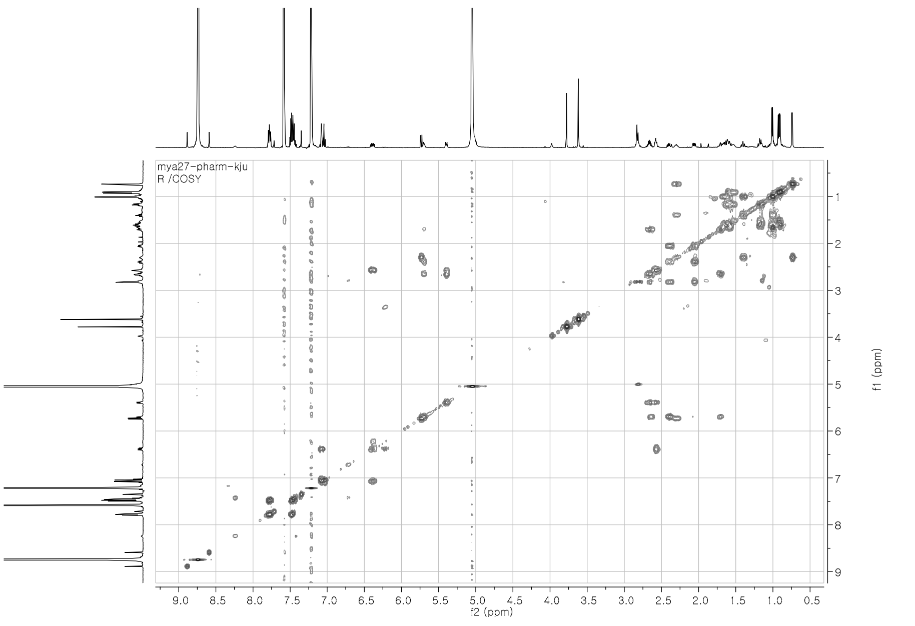

Figure S23. ^{1}H NMR spectrum of bis-*S*-MTPA ester of borrelidin D (**2a**) at 600 MHz in pyridine-d_5.

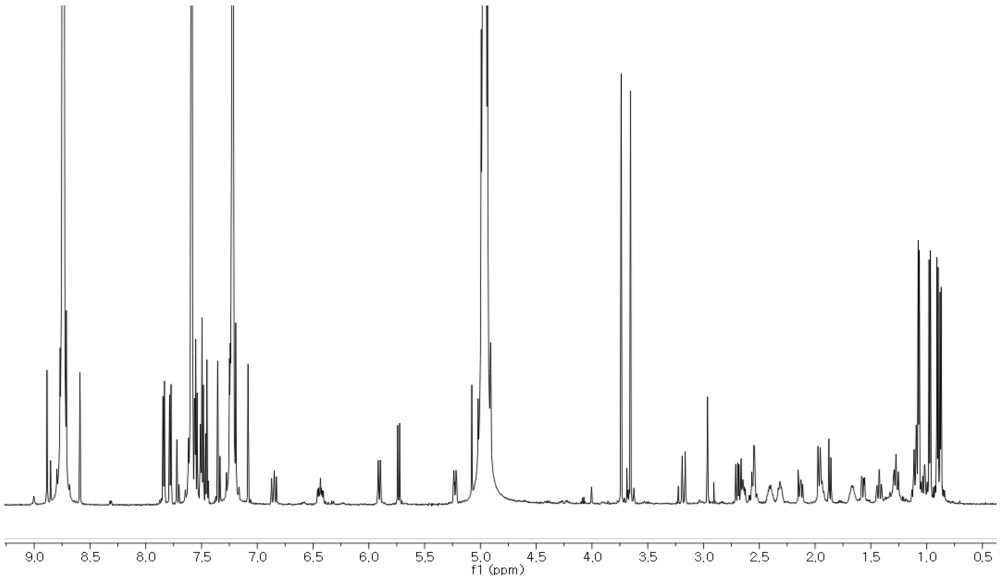

Figure S24. COSY NMR spectrum of bis-*S*-MTPA ester of borrelidin D (**2a**) at 600 MHz in pyridine-d_5.

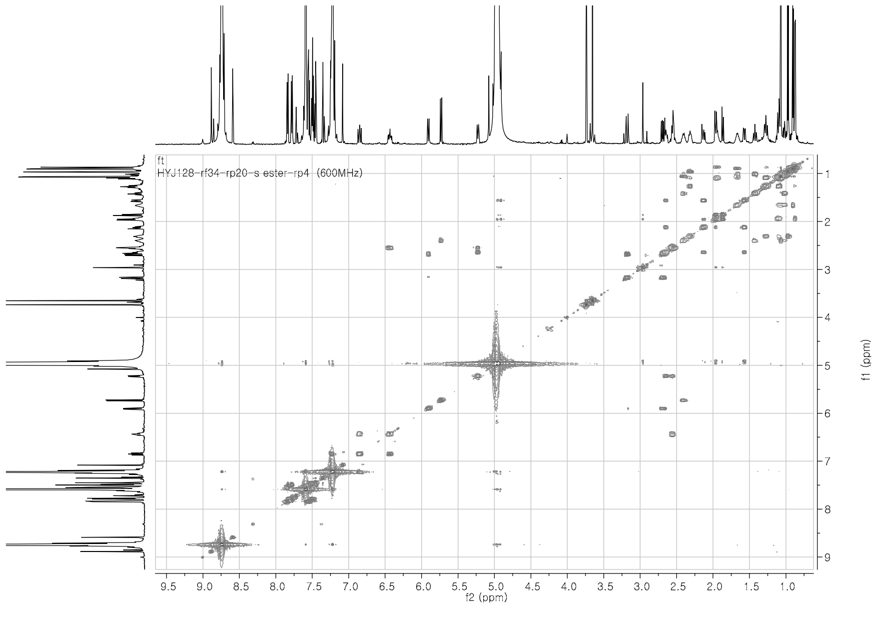

Figure S25. ^{1}H NMR spectrum of bis-*R*-MTPA ester of borrelidin D (**2b**) at 600 MHz in pyridine-*d*5.

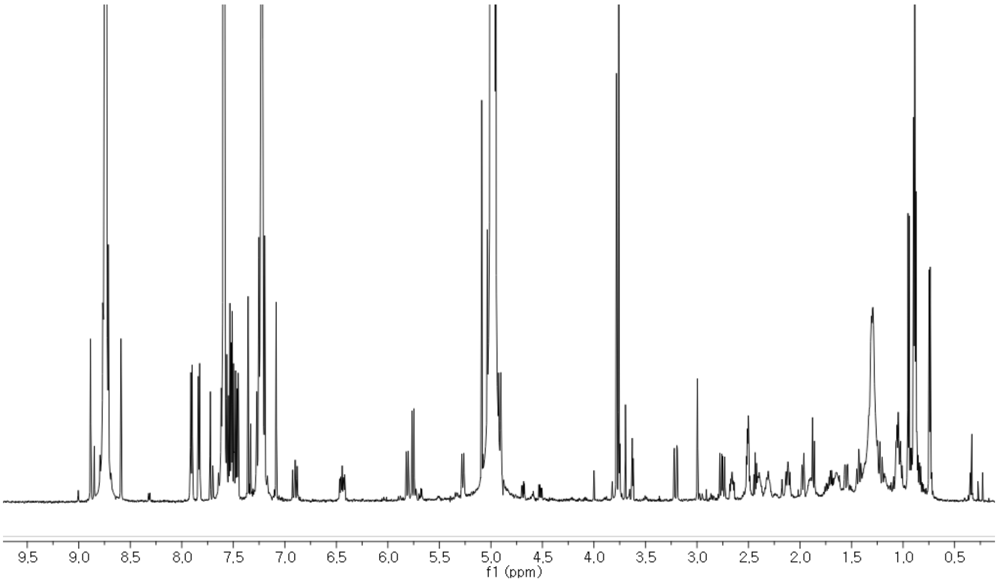

Figure S26. COSY NMR spectrum of bis-*R*-MTPA ester of borrelidin D (**2b**) at 600 MHz in pyridine-*d*5.

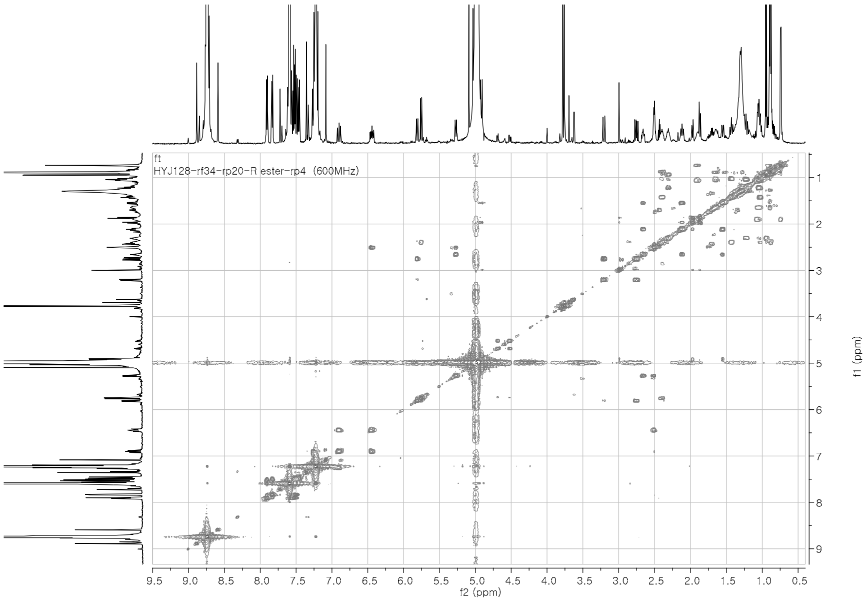

Figure S27. ^{1}H NMR spectrum of homo-decoupling ^{1}H experiment by irradiation ^{1}H at δ 2.38 of borrelidin E (**3**) at 600MHz in pyridine-*d*5.

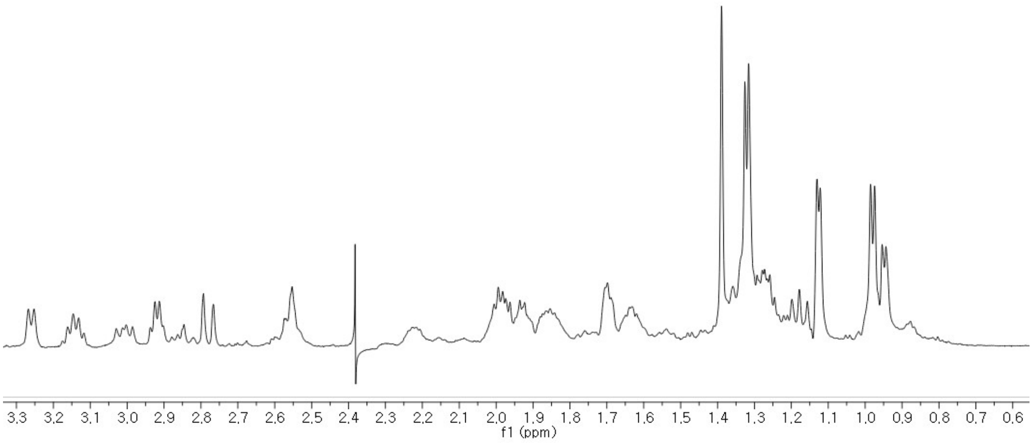

Figure S28. Experimental CD spectra of **1-4**.

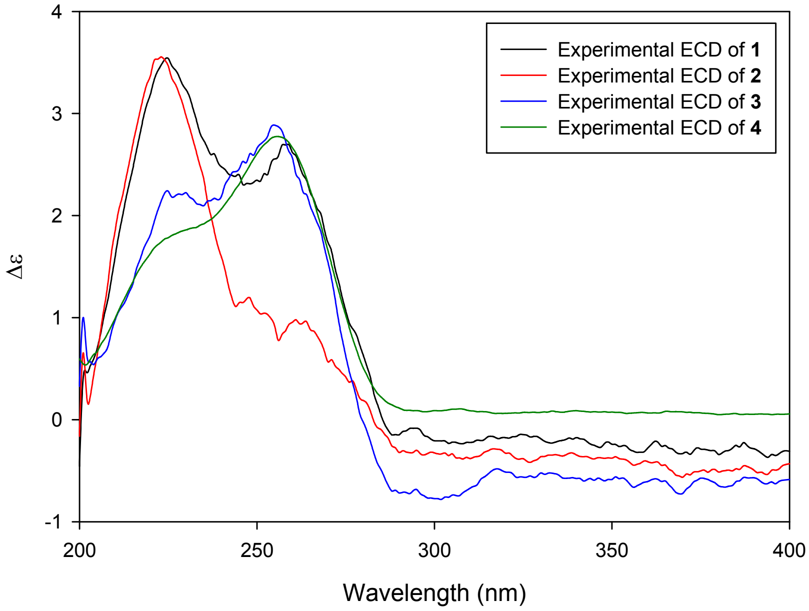

Supplement: Supplementary file 1 [file marinedrugs-15-00166-s001.pdf]
